# Absence of VGLUT3 Expression Leads to Impaired Fear Memory in Mice

**DOI:** 10.1523/ENEURO.0304-22.2023

**Published:** 2023-02-22

**Authors:** Camille de Almeida, Nida Chabbah, Camille Eyraud, Caroline Fasano, Véronique Bernard, Nicolas Pietrancosta, Véronique Fabre, Salah El Mestikawy, Stephanie Daumas

**Affiliations:** 1 Sorbonne Université, Institut National de la Santé et de la Recherche Médicale, Centre National de la Recherche Scientifique, Neuroscience Paris Seine – Institut de Biologie Paris Seine (NPS – IBPS), Paris 75005, France; 2 Douglas Mental Health University Institute, Department of Psychiatry, McGill University, Montréal QC H4H 1R3, Quebec, Canada

**Keywords:** aversive memories, pattern separation, spatial memory, vesicular glutamate transporter type 3

## Abstract

Fear is an emotional mechanism that helps to cope with potential hazards. However, when fear is generalized, it becomes maladaptive and represents a core symptom of posttraumatic stress disorder (PTSD). Converging lines of research show that dysfunction of glutamatergic neurotransmission is a cardinal feature of trauma and stress related disorders such as PTSD. However, the involvement of glutamatergic co-transmission in fear is less well understood. Glutamate is accumulated into synaptic vesicles by vesicular glutamate transporters (VGLUTs). The atypical subtype, VGLUT3, is responsible for the co-transmission of glutamate with acetylcholine, serotonin, or GABA. To understand the involvement of VGLUT3-dependent co-transmission in aversive memories, we used a Pavlovian fear conditioning paradigm in VGLUT3^–/–^ mice. Our results revealed a higher contextual fear memory in these mice, despite a facilitation of extinction. In addition, the absence of VGLUT3 leads to fear generalization, probably because of a pattern separation deficit. Our study suggests that the VGLUT3 network plays a crucial role in regulating emotional memories. Hence, VGLUT3 is a key player in the processing of aversive memories and therefore a potential therapeutic target in stress-related disorders.

## Visual Abstract

**Figure:**
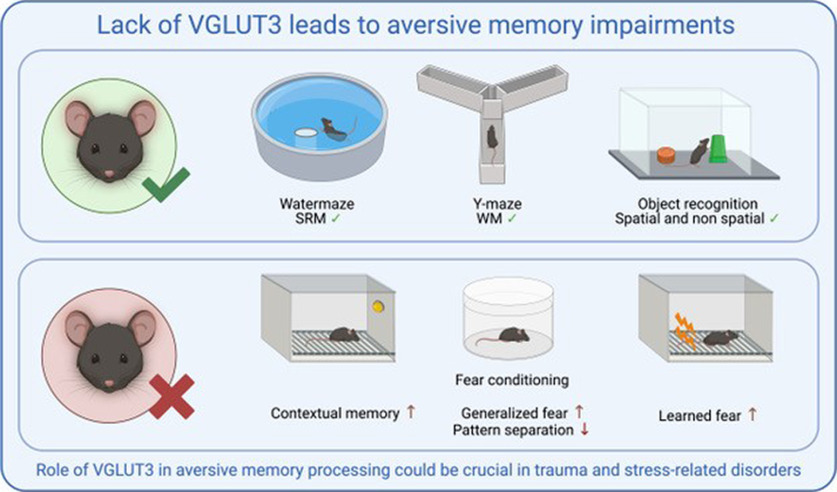

## Significance Statement

The development and maintenance of aversive memories play a central role in the adaptation of individuals to their environment. Structures such as the amygdala, the hippocampus or the basal forebrain are part of the neuronal basis of these memories. Recently, GABAergic, serotonergic and cholinergic neurons capable of releasing glutamate as well have been discovered within these structures. Glutamate is loaded into the vesicles of these neurons through the vesicular type 3 transporter, VGLUT3. Here we use mice lacking VGLUT3 to study the role of this glutamatergic cotransmission in the establishment and maintenance of fear memory in mice, thereby providing insight into its fine-tuning and paving the way to the development of new therapeutic targets.

## Introduction

Fear is an emotion in response to a threat that is essential for survival. However, generalization of fear is a core symptom of major psychiatric disorders such as anxiety disorders, phobia, panic disorder, and posttraumatic stress disorder (PTSD; Lissek and van Meurs, 2015; Besnard and Sahay, 2016). Major progress has been made thanks to animal studies of aversive memories through the Pavlovian fear conditioning paradigm (LeDoux, 2012). This test consists of associating an initially neutral stimulus (such as a tone, a light, or a context) to an aversive event (such as a footshock; Maren et al., 2013). This paradigm is widely used to dissect mechanisms underlying fear learning and memory, and to better understand fear-related disorders.

Fear generalization is defined by the fact that a distinct, but perceived by the animal as similar, context elicits fear responses. The balance between contextual discrimination and generalization is a crucial aspect of the expression of fear. Fear generalization is currently considered a central feature of generalized anxiety and PTSD (Lissek, 2012; Mahan and Ressler, 2012).

An effective treatment for fear generalization is based on extinction training to reduce acquired fear (Rothbaum and Davis, 2003; Craske et al., 2008). Fear extinction consists of new inhibitory learning after repeated presentations of fear-associated stimulus, in the absence of the aversive event, leading to a gradual decrease in the magnitude of the fear response (Myers and Davis, 2007). However, after extinction fear memory is not erased, but inhibited, as it can reappear in spontaneous recovery, external disinhibition, renewal, and reinstatement (Maren and Holmes, 2016). Therefore, it is important to better characterize neural circuits underlying the formation and maintenance of aversive memories if we want to understand and treat generalized fear more efficiently.

The neuronal circuits and the neuromodulators regulating emotional memories are well characterized. Emotional memories rely on a complex network including the amygdala, the hippocampus and the prefrontal cortex (Tovote et al., 2015). The amygdala is necessary for fear processing from acquisition to expression, whereas the hippocampus is mainly involved in contextual memory processing (Fanselow, 2000; Myers and Davis, 2007; Sierra-Mercado et al., 2011; Marek et al., 2018). Finally, the infralimbic (IL) and the prelimbic areas of the prefrontal cortex are essential for fear extinction (Sierra-Mercado et al., 2011; Marek et al., 2019).

Several studies have highlighted the involvement of neurotransmitters including glutamate, GABA, acetylcholine and serotonin signaling in fear processing (Craske et al., 2008; Christianson et al., 2010; Johnson et al., 2015; Ballinger et al., 2016; Baratta et al., 2016; Jiang et al., 2016; Knox, 2016; Wilson and Fadel, 2017; Krabbe et al., 2018). Interestingly several subpopulations of neurons and fibers of the amygdala, the hippocampus or the prefrontal cortex release more than one neurotransmitter (for review, see El Mestikawy et al., 2011; Trudeau and El Mestikawy, 2018). Most of these bilingual neurons in the fear circuit express the atypical vesicular glutamate transporter type 3 (VGLUT3; Herzog et al., 2004; Amilhon et al., 2010; Omiya et al., 2015; Fasano et al., 2017; Rovira-Esteban et al., 2017; Sengupta and Holmes, 2019). Studies have illustrated the involvement of VGLUT3 neurons in psychiatric disorders (Sakae et al., 2015; Favier et al., 2020). Several studies have demonstrated that the absence of VGLUT3 in VGLUT3 neurons led to the abolishment of glutamatergic currents mediated by mGlu receptors in the striatum or the hippocampus (Sakae et al., 2015; Fasano et al., 2017; Favier et al., 2020) whereas others showed the abolition of a glutamatergic ionotropic currents (Varga et al., 2009; Higley et al., 2011). Interestingly, VGLUT3^–/–^ mice show a persistent hyper-reactivity to stress (Amilhon et al., 2010) and a dysregulation of their hypothalamic-pituitary-adrenal (HPA) axis (Balázsfi et al., 2018), but only a few studies focused on the role of VGLUT3 in the regulation of emotion and fear. A couple of studies previously showed that VGLUT3-deficient mice have a higher contextual fear memory and tend to generalize their fear to unrelated situations (Balázsfi et al., 2018) with no major other memory deficits (Fazekas et al., 2019).

In this context, our aim was to confirm the role of VGLUT3 in aversive memories and to deepen our understanding of it by using a combination of behavioral paradigms. Using a Pavlovian fear conditioning paradigm, we report that VGLUT3^–/–^ mice express more stable and generalized contextual memories associated with a deficit of pattern separation. Interestingly, VGLUT3^–/–^ mice have no deficit in nonaversive learning or in working memory (WM), spatial reference memory (SRM), or in recognition memory. These results highlight the specific role of the VGLUT3-positive network in the establishment and maintenance of aversive memories and most notably in the generalization of fear. They also provide evidence that VGLUT3 could be considered as a potential target for the treatment of stress-related disorders.

## Materials and Methods

### Animals

Animal care and experiments were conducted in accordance with the European Communities Council Directive for the Care and the Use of Laboratory Animals (86/809/EEC) and in compliance with the French Ministère de l’Agriculture et de la Forêt, Service Vétérinaire de la Santé et de la Protection Animale (authorization #01482.01 from ethics committee Darwin #5). All efforts were made to minimize the number of animals and to ensure their well-being. Animals were group caged and housed in a temperature-controlled room (20 ± 2°C) with free access to water and food under a 12/12 h light/dark cycle (light 7:30 A.M. to 7:30 P.M.).

VGLUT3^–/–^ mice (Gras et al., 2008) were on a C57BL6/J background. Heterozygous mice were bred to obtain VGLUT3^–/–^ mice and wild-type (VGLUT3^+/+^) littermates. Pups were weaned around 22 d old, marked by ear punch and genotyped using the ear sample. Experiments were performed with two- to four-month-old mice (159 males and 50 females). Animals were randomly allocated to experimental groups and investigators were blinded for experimental procedures. Total animal number used in each paradigm is presented in Table 1.

**Table 1:** Cohorts used

Experiment (figure)	Sex	*N* of VGLUT3^+/+^ (WT)	*N* of VGLUT3^−/−^ (KO)
Watermaze 22°C (Fig. 1*D–F*)	F	15	11
Watermaze 19°C (Fig. 1*G–I*)	F	13	11
Object recognition (Fig. 2)	M	13	12
Shock sensitivity (Fig. 3*A*)	M	8	6
Fear conditioning (Fig. 3*B–F*)	M	12	12
Pattern separation (Fig. 4)	M	11	10
Immediate shock (Fig. 5)	M	14	20
Fear extinction (Fig. 6)	M	12	12
Y maze (Fig. 7)	M	9	8
Total		107	102

### Behavioral paradigms

#### The watermaze task

The WM test was performed as described previously (Daumas et al., 2008). The mice were monitored with a video tracking system (AnyMaze). First mice went through a 4-d cuetask protocol where the 1.8-m diameter pool is surrounded with curtains, and a cue placed on the platform (60-s trials, four trials a day, Inter-Trial-Interval (ITI) = 20 min). For the spatial reference memory (SRM) task, the platform was centered in one of the four quadrants and kept stable throughout the task (without any cue on it). The protocol lasted 5 d (90-s trials, four trials a day, ITI = 10 min). Ten minutes after the last trial on day 5, a 60-s probe test (SRM-10 min) was conducted during which the platform was removed. In order to avoid extinction, an additional trial with the platform was done immediately after each probe test. A second probe test was performed 72 h after assessing the long-term memory of the mice (SRM-72 h). For the SRM Reversal (SRM-R) task, which was conducted immediately after the second probe test, learning flexibility was assessed by moving the platform to the opposite quadrant used for the SRM task. The animals were trained for 3 d (90-s trials, four trials a day, ITI = 10 min) and spatial memory was assessed at 10 min (SRM-R-10 min) and 72 h (SRM-R-72 h) after the last SRM-R trial. Data for the following parameters were collected: latency to reach the platform location, path length, swim speed, thigmotactic behavior and the percentage of time spent in the quadrant zones.

#### Novel place recognition/novel object recognition

The novel place recognition (NPR)/novel object recognition (NOR) task was performed in a square open-field (25 cm) with sawdust on the floor and cues on the walls. Habituation consisted of (1) a 10-min exploration period of the open-field with cagemates (day 1), (2) two 5-min periods during which each mouse was placed individually in the empty open-field on two consecutive days (days 2–3), and (3) a 5-min period during which the mice were placed in the open-field with two identical objects (day 4). On the training day (day 5), mice were allowed to explore two new identical objects until they had accumulated 15 s then 10 s of total inspection time during the first and second training session, respectively. Since VGLUT3^–/–^ mice are more anxious, the protocol was adapted in this way rather than a fixed 10 min training session, to ensure that all animals explore the objects sufficiently to establish memory formation. Therefore, the length of the session was different between animals, but the exploration of the objects was identical. On day 6, the mice were tested for the NOR paradigm (10 min) during which one of the original objects was replaced with a new object. On day 7 we started the NPR paradigm during which two new objects were placed in the open-field. As for NOR, 2 sessions of training were run and consisted in accumulating 15 and 10 s of total exploration time. Twenty-four hours later (day 8), the mice were tested in the NPR paradigm (10 min): the same pair of familiar objects was used but one of the objects was displaced in another corner of the open- field. The percentage of time exploring the new object was calculated as a discrimination index: [novel/(novel + familiar)].

#### Y maze

Working memory was assessed with a Y maze apparatus (Imetronic). Mice freely explored the maze for 10 min. The total number of entries was counted as well as the spontaneous alternation. Spontaneous alternation occurs when a mouse enters a different arm of the maze three consecutive times. The percentage of spontaneous alternation was calculated by dividing the number of spontaneous alternations by the total number of arm entries minus 2 and multiplied by 100.

#### Fear conditioning experiments

The Fear Conditioning Apparatus (BIOSEB) is made of black methacrylate walls, a grid floor and transparent ceiling and front door. Panlab software (BIOSEB) was used to carry out the experiments and record freezing behavior. A video recording system (Multimedia Video Record) allowed manual scoring of freezing levels to validate the automatic counts.

##### Shock sensitivity paradigm

Because VGLUT3 is present in peripheral sensory neurons and contributes to mechanical pain (Seal et al., 2009), we assessed the sensitivity to electric footshocks in VGLUT3^–/–^ mice. A train of electric footshocks (ES, 1-s duration) was delivered starting from 0.1 mA and gradually increasing by 0.05 mA every 30 s. Shock delivery was stopped when all expected behavioral responses were observed: increased locomotor activity (movement), vocalization, running and jumping. The intensity of the electric shock that first triggered each of these behaviors was recorded.

##### Fear conditioning paradigm

The fear conditioning paradigm was used to study learning and memory of aversive stimuli as previously reported (LeDoux, 2003; Daumas, 2005). Since VGLUT3^–/–^ mice are deaf (Ruel et al., 2008; Seal et al., 2008), a flashing light was used as the conditioned stimulus (CS: 20 s, 2 s ON/2 s OFF, 80 lux) and a 0.25-mA electric footshock as the unconditioned stimulus (US, 2 s).

After 3 d of habituation (6 min/d), the conditioning session took place on day 4. After 2 min in the chamber, the CS was triggered and its final 2 s coincided with the US. After a 30-s interval, a second CS-US pairing was presented. Memory tests were done on day 5. Contextual memory was assessed with the contextual test, and cue memory was assessed by the cue test 2 h later. For the contextual test, mice were placed in the conditioning context for 6 min without CS (light) or US. The cue test consisted of 3 min of exploration of a modified context (color, shape, light, and odor), followed by four CS presentations with an intertrial interval of 30 s.

##### Pattern separation

A pattern separation protocol was conducted for 11 d in two highly similar contexts: the shock associated context A and the safe context B as described by (Sahay et al., 2011). On day 0, mice were introduced into context A and after 185 s received a 0.75 mA US for 2 s. During the following 10 d, mice were exposed to the US-associated context A (183-s exploration–2-s US–15-s exploration, before being removed to home cage) and 1 h later to the safe context B (180-s exploration) in a defined order. Freezing behavior was assessed during the first 180 s for each context.

##### Immediate shock procedure

Mice were submitted to a no shock (NS) or an immediate shock (IS) procedure. For the NS, mice were free to explore the conditioning cage for 30 s. In the IS procedure, mice received an immediate shock (0.25 mA, 2 s) immediately after their placement in the conditioning chamber and were removed after 30 s. Generalized fear was evaluated 24 h later by placing the animals in the conditioning chamber (same context; SC) or in a novel box (novel context; NC) for 5 min.

##### Fear extinction learning

Fear extinction learning and memory were studied for 15 d. Mice were habituated to the conditioning chamber for 2 min before ten CS-US were delivered at 75 s intervals. From day 2 to day 8, extinction took place in the modified context. Mice were exposed to 10 presentations of CS with an interval of 85 s under red light illumination. A learning index (LI) was calculated daily. This index is used to ascertain the daily extinction rate by calculating the difference between the first and last CS-induced freezing. On day 15, mice were re-exposed to the conditioning context with ten CS presentations to assess fear recall. On day 18, they were placed in a new context and ten CS were once again presented to evaluate renewal in a new context.

#### Statistics

Statistical comparisons were performed with Prism 9 (GraphPad software Inc. for macOS). Each statistical test was appropriately chosen for the relevant experimental design. Sidak’s multiple comparisons test was performed for *post hoc* analysis when required unless otherwise indicated. All data are presented as the mean ± SEM, with differences considered significant at *p* < 0.05. Complete analysis and statistics are presented in Extended Data Figures 1-1, 2-1, 3-1, 4-1, 5-1, 6-1, 7-1.

## Results

Fear conditioning is based on learning/memory and on the propensity of mice to feel and react to electric footshock. VGLUT3 is expressed in the hippocampus where it contributes to hippocampal plasticity and network properties (Fasano et al., 2017). On the other hand, VGLUT3 is also found in subsets of neurons in pain circuits (Landry et al., 2004; Seal et al., 2009; Draxler et al., 2014; Peirs et al., 2015; Larsson and Broman, 2019; Sakai et al., 2020). Therefore, before using the fear conditioning paradigm, we assessed learning, spatial memory and pain threshold (i.e., response to foot shock) in VGLUT3^–/–^ mice.

### The absence of VGLUT3 does not impair learning and memory in mice

**Figure 1.**
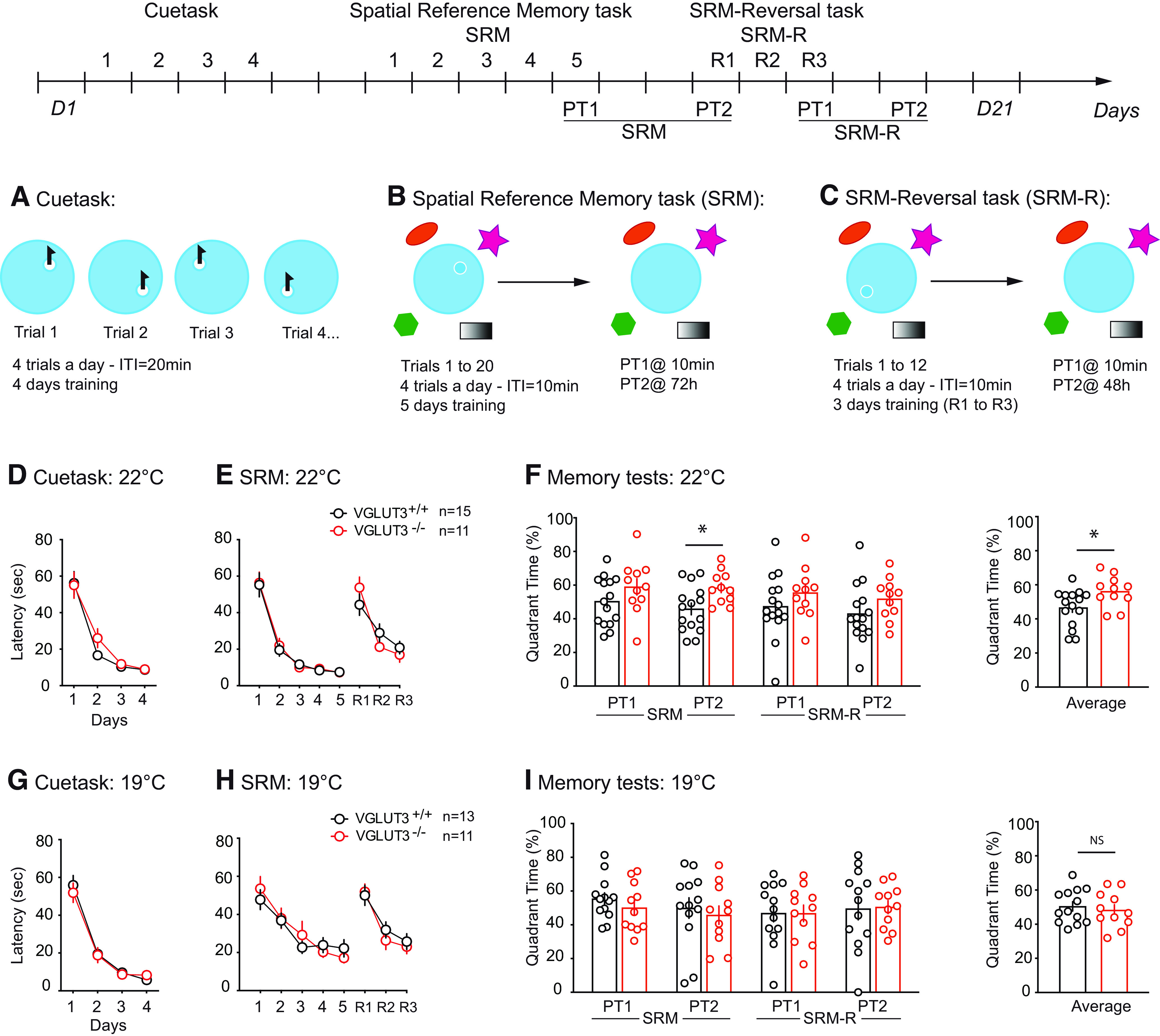
Cue and spatial reference memory in VGLUT3^–/–^ mice. ***A–C***, Watermaze experimental design: (***A***) Cuetask, (***B***) SRM task, and (***C***) SRM-reversal task. ***D–F***, Mice were trained in 22°C water. VGLUT3^–/–^ mice showed no deficit of learning either in the cue (***D***), or the SRM and reversal (***E***), tasks. ***F***, Memory assessment was performed 10 min (PT1 for SRM and SRM-R), 72 h (PT2 for SRM), and 48 h (PT2 for SRM-R) after training, and VGLUT3^–/–^ mice showed better performance at 72 h after training, and in the PT average. ***G–I***, Mice were trained in water at 19°C. VGLUT3^–/–^ mice show no deficit of learning either in the Cue (***G***), or the SRM and reversal (***H***) tasks. ***I***, No differences were observed in memory tests done at different times or on average. Data are mean ± SEM. Differences between genotypes: *p < 0.05. PT: probe test; R: reversal; NS: non significant. All corresponding statistics are presented in Extended Data Figure 1-1.

To explore the consequences of VGLUT3 deletion on spatial and nonspatial memories, we first used the watermaze task (WM; Fig. 1; statistics details can be found in Extended Data Fig. 1-1). Relative to wild-type littermates, VGLUT3^–/–^ mice displayed no impairment of learning in either the nonspatial (Fig. 1*D*) or the spatial (Fig. 1*E*) task. We observed a main effect of time but no main effect of genotypes or interaction between time and genotype. Therefore, both genotypes improved their learning during the training days (Fig. 1*D*,*E*, *p* < 0.0001). To challenge them and assess their learning flexibility, a 3-d reversal task was performed immediately after the spatial reference learning task (Fig. 1*E*, R1–R3). On day 1 of reversal learning (Fig. 1*E*, R1) both groups increased their latency to reach the new platform location, and then similarly improved their performance (time: *p* < 0.0001, genotype: *p* = 0.882; Fig. 1*E*). Spatial memory was assessed 10 min and 72 h after training completion for SRM and SRM-R. In all tested conditions, control littermates as well as VGLUT3^–/–^ mice spent significantly more than 25% of probe trial time in the targeted quadrant, indicating intact spatial reference memory (group performance vs 25% *p* < 0.05; Fig. 1*F*). However, during the long-term memory test, VGLUT3^–/–^ mice showed better performances (SRM-PT2) than controls (Fig. 1*F*). Since VGLUT3^–/–^ mice are more vulnerable to anxiety than WT mice (Amilhon et al., 2010), we explored the contribution of anxiety to memory formation and learning in VGLUT3^–/–^ mice in a more stressful condition, when the water temperature was lowered to 19°C (Sandi et al., 1997). At 19°C, we observed no main effect of genotype or interaction between genotype and time, but a main effect of time for both cuetask and SRM/SRM-R (Fig. 1*G*,*H*). A three-way ANOVA revealed no main effect of genotype, tests or water temperature and no interactions between these parameters except for the temperature × genotype (*p* = 0.04; see Extended Data Fig. 1-1 for statistical details). Moreover, in all tested conditions, VGLUT3^–/–^ and control mice show similar performances and spent >25% of their time in the correct quadrant (Fig. 1*I*). These data show no deficit of learning and memory in VGLUT3^–/–^ mice in the WM paradigm.

10.1523/ENEURO.0304-22.2023.f1-1 Extended Data Figure 1-1 Statistics for watermaze experiments. 1: SRM 10 min; 2: SRM 72 h; 3: SRM-R 10 min; 4: SRM-R 48 h; 5: PTs average. Download Figure 1-1, DOCX file.

**Figure 2.**
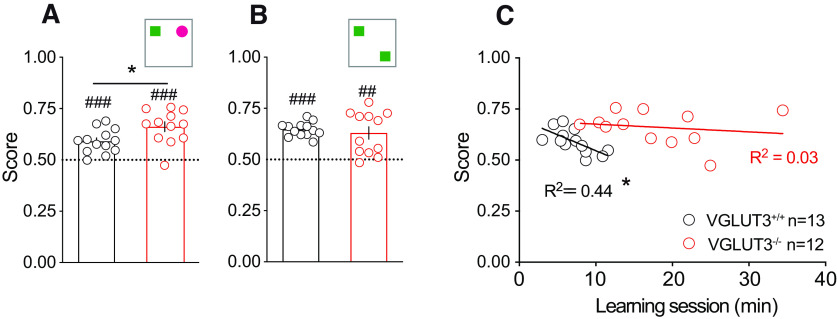
Object and spatial recognition in VGLUT3^–/–^ mice. ***A***, Object recognition (OR): both groups show OR memory, with VGLUT3^–/–^ mice having higher scores. ***B***, Spatial recognition (SR): both groups show comparable SR memory level. ***C***, There is no correlation between learning sessions duration and VGLUT3^–/–^ mice performances in OR. Slopes are −0.01563 for WT and −0.001889 for VGLUT3^–/–^ mice. Data are mean ± SEM. Differences between genotypes: *p < 0.05. Differences to chance level: ##*p* < 0.01, ###*p* < 0.001. All corresponding statistics are presented in Extended Data Figure 2-1.

We then studied spontaneous learning and memory using the object recognition paradigm. We observed for both genotypes a significant difference from chance level (score 0.5) revealing long-term recognition memory for objects (Fig. 2*A*; All corresponding statistics are presented in Extended Data Figure 2-1) and position (Fig. 2*B*) in all animals. VGLUT3^–/–^ mice show higher performances than control littermates in the object recognition task (Fig. 2*A*). Since VGLUT3^–/–^ mice spent significantly more time in the open field, we wondered whether the time spent during training was correlated with the memory score obtained in the object recognition test. The correlation curve (Fig. 2*C*) and the linear regression revealed no correlation between memory score and the length of the session in VGLUT3^–/–^ mice (*R*^2^ = 0.03, *F*_(1,11)_ = 0.31, *p* > 0.05; equation: Y = −0.001889*X + 0.6951). These experiments do not reveal major learning or memory impairment in VGLUT3^–/–^ mice.

10.1523/ENEURO.0304-22.2023.f2-1 Extended Data Figure 2-1 Statistics for object recognition experiments. Download Figure 2-1, DOCX file.

### Footshock sensitivity is not altered by VGLUT3 deletion

**Figure 3.**
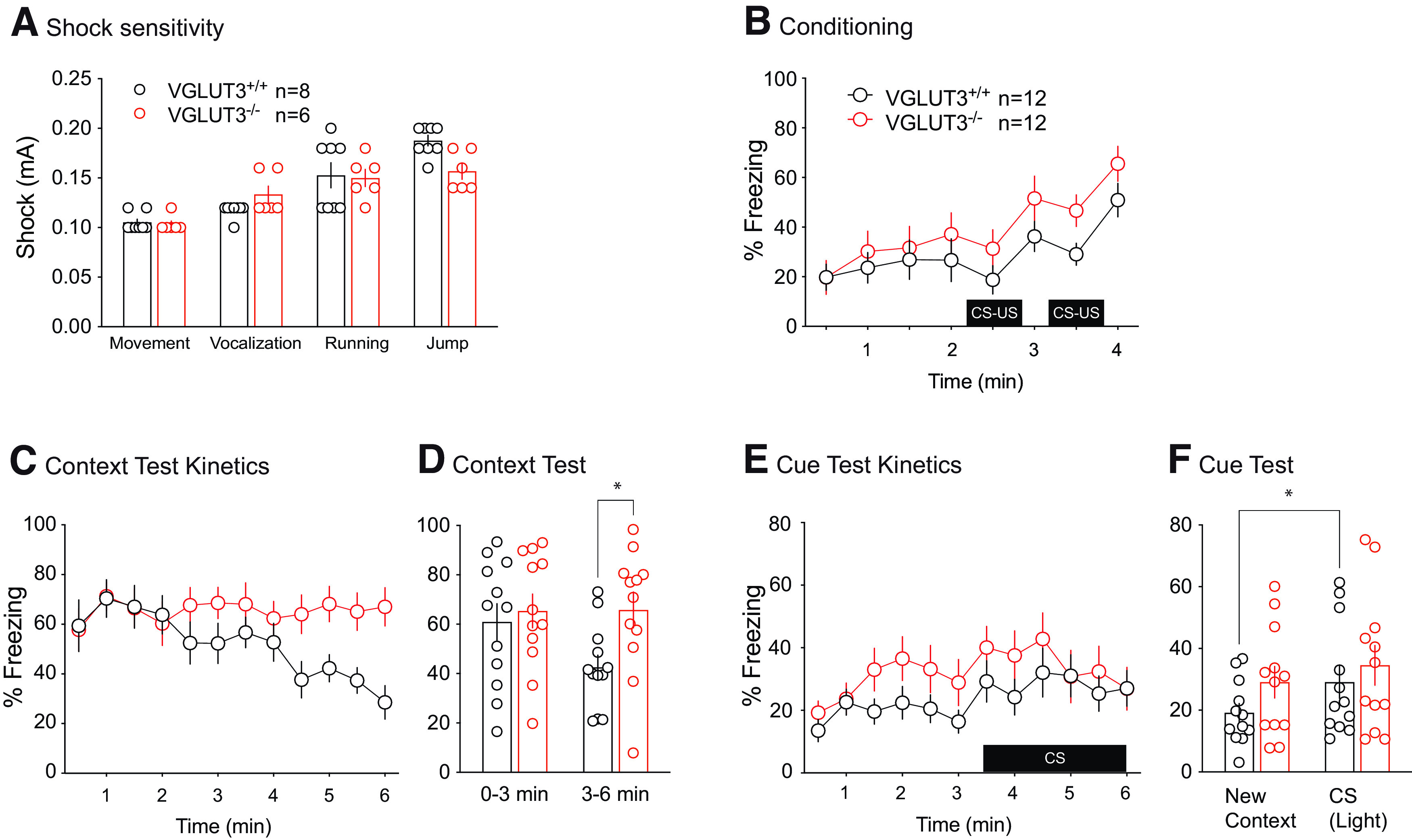
Contextual and cued fear memories of VGLUT3^–/–^ mice. ***A***, Shock sensitivity assessment, i.e., the intensity for which the mice express a given behavior (movement, vocalization, running, or jump). ***B–F***, Fear memories in VGLUT3^–/–^ mice. ***B***, Freezing levels during fear conditioning consisting of two CS-US pairings. ***C***, ***D***, Contextual memory was tested 24 h after conditioning and revealed a more stable memory in VGLUT3^–/–^ mice. ***E***, ***F***, Cued memory test revealed high level of freezing to new context for VGLUT3^–/–^ mice. Data are mean ± SEM *post hoc* comparisons: *p < 0.05. All corresponding statistics are presented in Extended Data Figure 3-1.

Deletion of VGLUT3 did not affect the behavioral responses (movement, vocalization, running, jump) elicited by footshock stimuli of varying intensity (Fig. 3*A* and statistics in Extended Data Fig. 3-1). This result shows that pain sensitivity to electric footshocks is unaffected in VGLUT3^–/–^ mice.

10.1523/ENEURO.0304-22.2023.f3-1 Extended Data Figure 3-1 Statistics for fear conditioning experiments. Download Figure 3-1, DOCX file.

### Visual and contextual fear conditioning are altered in the absence of VGLUT3

During conditioning we observed no main effect of genotype, no interaction between genotype and time, only a main effect of time (Fig. 3*B*; All corresponding statistics are presented in Extended Data Figure 3-1). We then assessed contextual memory 24 h after conditioning (Fig. 3*C*,*D*). The time course analyses of the freezing rate during the 6 min test shows an interaction and a time effect, but no main effect of genotype (Fig. 3*C*). When we analyzed the test by 3 min bins (Fig. 3*D*), a clear genotype difference arises. *Post hoc* analysis revealed higher freezing rate in VGLUT3^–/–^ than in VGLUT3^+/+^ mice in the last 3 min of the test (0–3 min: *t*_(44)_ = 0.4722, *p* = 0.87; 3–6 min: *t*_(44)_ = 2.464, *p* = 0.03; Sidak’s multiple comparisons test; Fig. 3*D*). The cue test was then done by exposing mice to the flashing light in a novel environment. Mice were free to explore the new context for 3 min before the light (CS) was triggered (Fig. 3*E*,*F*). The global analysis reveals only a main effect of time but no main effect of genotype or interaction between genotype and time (Fig. 3*E*; All corresponding statistics are presented in Extended Data Figure 3-1). Remarkably, the freezing rate significantly increased in VGLUT3^+/+^ mice but not in VGLUT3^–/–^ mice after CS presentation in the new context (respectively, *t*_(22)_ = 2.541, *p* = 0.03; and *t*_(22)_ = 1.395, *p* = 0.32; Sidak’s multiple comparisons test; Fig. 3*F*). One possible explanation of the higher fear expression observed in VGLUT3^–/–^ mice in the new context could be that once conditioned, they show a higher fear response to a new context with either no specific freezing responses associated with the US or too low to be observed.

### The absence of VGLUT3 leads to a deficit in pattern separation

**Figure 4.**
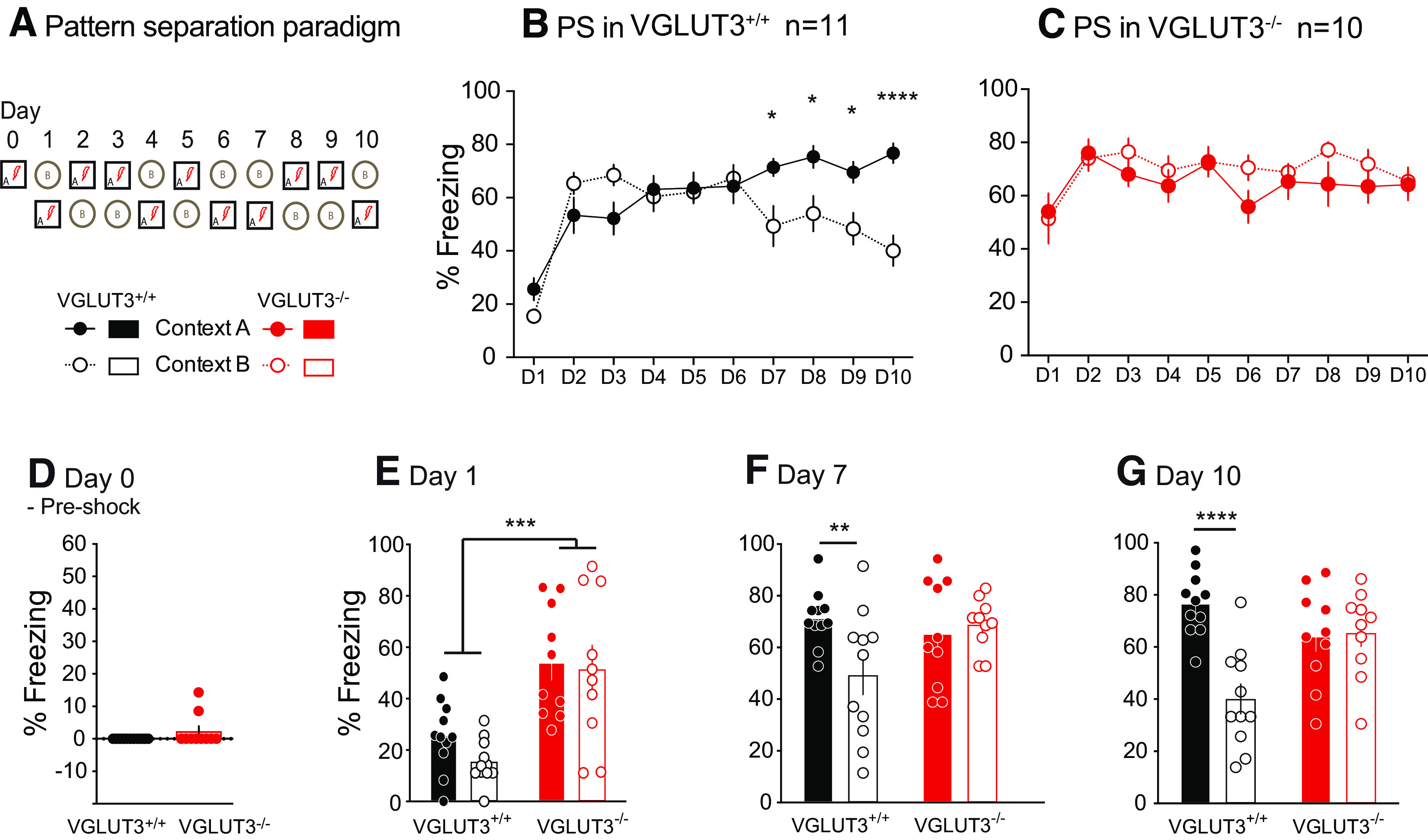
Pattern separation of VGLUT3^–/–^ mice. ***A***, Behavioral protocol. ***B***, VGLUT3^+/+^ mice performances. ***C***, VGLUT3^–/–^ mice performances. ***D–G***, Freezing levels on different days: (***D***) day 0, before conditioning; (***E***) day 1, VGLUT3^–/–^ mice already show a higher freezing level; (***F***) day 7, VGLUT3^+/+^ mice start to discriminate the different contexts. ***G***, On day 10, VGLUT3^–/–^ mice still do not discriminate the different contexts. Data are mean ± SEM, *p < 0.05, **p < 0.01, ***p < 0.001, ****p < 0.0001. All corresponding statistics are presented in Extended Data Figure 4-1.

Cued memory alterations in VGLUT3^–/–^ mice (Fig. 3*E*,*F*) might be caused by a deficit to discriminate between the two contexts, that associated with an US versus the safe one, a process governed by pattern separation. To examine this possibility, we submitted a group of mice to a pattern separation protocol (Fig. 4*A*) where context A is always associated with an electric shock (ES), whereas context B is safe and free of ES. In VGLUT3^+/+^ mice, we observed no main effect of context, but a main effect of time and an interaction between context and time (Fig. 4*B*; Extended Data Fig. 4-1) Over time VGLUT3^+/+^ mice learn to dissociate the two contexts since they significantly freeze less from day 7 to day 10 (day 7, *t*_(11)_ = 3.031, *p* = 0.02; day 8, *t*_(11)_ = 2.933, *p* = 0.03; day 9, *t*_(11)_ = 2.917, *p* = 0.03; day 10, *t*_(11)_ = 5.038, *p* < 0.0001; Sidak’s multiple comparisons test; Fig. 4*B*). Strikingly, in VGLUT3^–/–^ mice we observed no main effect of context, or interaction between context and time but a main effect of time (Fig. 4*C*; Extended Data Fig. 4-1). VGLUT3^–/–^ mice did not learn to discriminate the two contexts as high freezing levels were maintained over the 10 d of the test (Fig. 4*C*). Furthermore, VGLUT3^–/–^ mice showed comparable levels of spontaneous freezing on day 0 before the occurrence of the first ES (Fig. 4*D*).

10.1523/ENEURO.0304-22.2023.f4-1 Extended Data Figure 4-1 Statistics for pattern separation experiment. Download Figure 4-1, DOCX file.

However, on day 1, after conditioning, we observed a main effect of genotype, but no main effect of context or interaction between context and genotype (Fig. 4*E*; Extended Data Fig. 4-1). On days 7 and 10, we observed no main effect of genotype, but a main effect of context and an interaction between context and genotype (Fig. 4*F*,*G*; Extended Data Fig. 4-1). VGLUT3^+/+^ mice clearly dissociated context A from B [day 7, *t*_(11)_ = 4.04, *p* = 0.001 (Fig. 4*F*); day 10, *t*_(11)_ = 7.934, *p* < 0.0001; Sidak’s multiple comparisons test (Fig. 4*G*)]. This was not the case with VGLUT3^–/–^ mice. Altogether, these results illustrate a deficit in pattern separation in VGLUT3^–/–^ mice.

### The absence of VGLUT3 leads to generalized fear after aversive experiences

**Figure 5.**
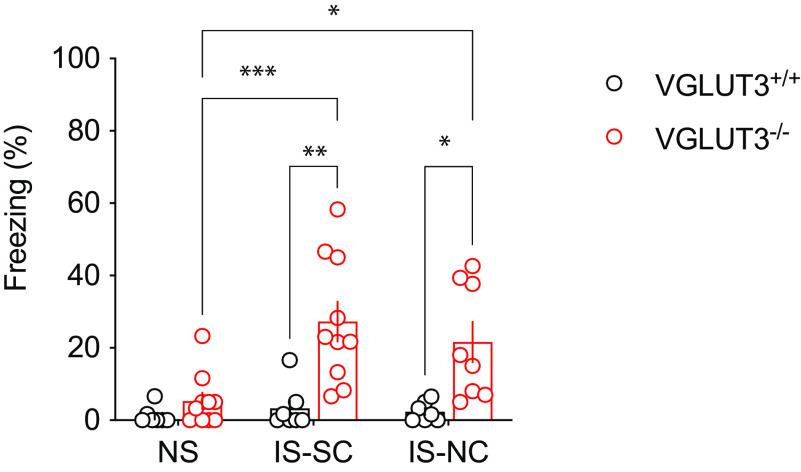
Immediate shock in VGLUT3^–/–^ mice. Mice were subjected to a no shock (NS) or immediate shock (IS) protocol to assess their levels of spontaneous freezing after experiencing an electric footshock. They were either tested in the same context (IS-SC) or in a novel context (IS-NC). WT mice did not show any freezing after either procedure, whereas VGLUT3^–/–^ mice expressed a significant increase of freezing behavior only after the IS, in either context. Data are mean ± SEM *post hoc* comparisons: *p < 0.05; **p < 0.01; ***p < 0.001. All corresponding statistics are presented in Extended Data Figure 5-1.

The observed deficit in pattern separation could also represent generalized fear in VGLUT3^–/–^ mice. To investigate this point, we submitted a group of VGLUT3^+/+^ mice and VGLUT3^–/–^ mice to an immediate shock paradigm (Fig. 5; All corresponding statistics are presented in Extended Data Figure 5-1). On day 1, mice were introduced to a context and either immediately received a footshock [immediate shock (IS) condition] or nothing [no shock (NS) condition]. The next day, they were tested in the same context (SC) or in a new context (NC). As expected, the immediate shock (IS) did not elicit freezing behavior on day 2 in VGLUT3^+/+^ mice, in either context (Fig. 5, IS-SC or IS-NC). VGLUT3^–/–^ mice showed no freezing when they were not shocked (Fig. 5, NS), however significant higher freezing levels were observed after the IS procedure in both contexts (Fig. 5, IS-SC and IS-NC). These results revealed increased freezing levels in VGLUT3^–/–^ mice after experiencing an aversive stimulus.

10.1523/ENEURO.0304-22.2023.f5-1 Extended Data Figure 5-1 Statistics for immediate shock experiments. NS: no shock; IS: immediate shock; SC: same context; NC: new context Download Figure 5-1, DOCX file.

### Visual fear extinction is altered in the absence of VGLUT3

**Figure 6.**
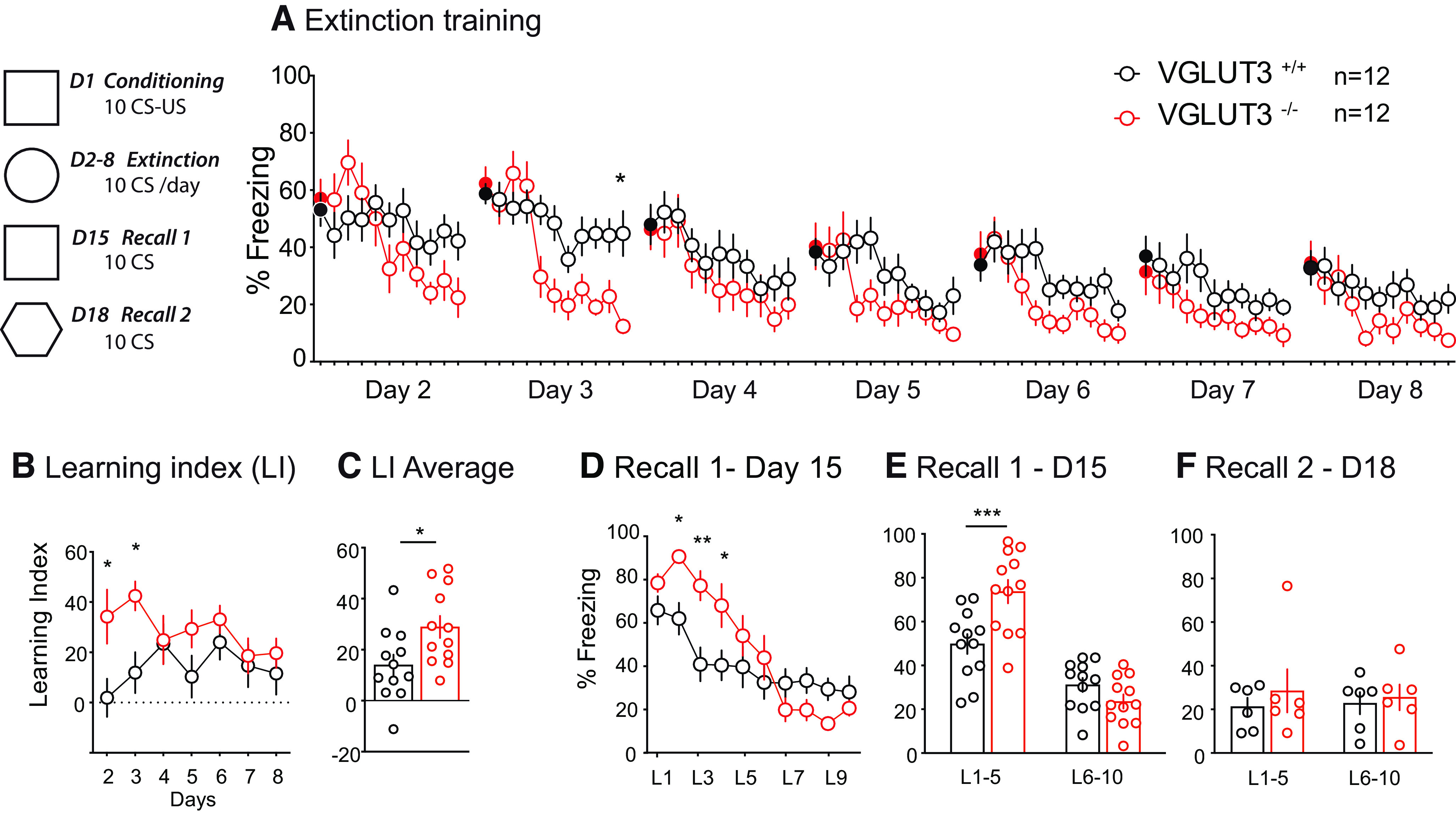
Extinction fear in VGLUT3^–/–^ mice. ***A***, Extinction learning over a 7-d period. Filled black and red circles represent the freezing levels of VGLUT3^+/+^ mice and VGLUT3^–/–^ mice, respectively, before the presentation of the first CS. Open circles are used for the 10 subsequent CS. ***B–E***, Fear memory in VGLUT3^–/–^ mice. ***B***, ***C***, The learning index (LI) was calculated to illustrate learning efficacy over time (***B***) and on average (***C***). ***D***, ***E***, Original memory was recalled on day 15. ***F***, On day 18, freezing to CS was assessed in a new hexagonal context. Data are mean ± SEM, *p < 0.05, **p < 0.01, ***p < 0.001. L: light (CS). All corresponding statistics are presented in Extended Data Figure 6-1.

Because of the impairment described in the cue-test (Fig. 3*E*,*F*), we wondered whether VGLUT3^–/–^ mice were not fully conditioned with a discrete CS such as a light. To answer this question, a cue fear conditioning extinction protocol was performed (Fig. 6). On day 1, mice were exposed to 10 CS-US presentations in a square context, followed from day 2 to day 8 to a daily session of 10 CS-only presentations in a round context, to assess cue extinction (Fig. 6*A*). The overall analysis suggested a tendency for a main effect of genotype with a clear main effect of time and an interaction between time and genotype (Table 7). On day 3, both groups started the test with an equivalent high level of freezing that progressively decreased, reaching significance on the 10th CS presentation (*t*_(12)_ = 3.77, *p* = 0.01; Sidak’s multiple comparisons test; Fig. 6*A*).

10.1523/ENEURO.0304-22.2023.f6-1 Extended Data Figure 6-1 Statistics for fear extinction experiments. Download Figure 6-1, DOCX file.

To determine the extinction of learning performances of mice, we calculated a learning index (LI; Fig. 6*B*,*C*). We observed a main effect of genotype with no effect of time or interaction between time and genotype (Table 7). VGLUT3^–/–^ mice demonstrated a higher LI than VGLUT3^+/+^ mice during the first 2 d of the test, followed by a similar pattern for the two genotypes during days 4–8 (day 2: *t*_(12)_ = 2.922, *p* = 0.02; day 3, *t*_(12)_ = 2.761, *p* = 0.04; Sidak’s multiple comparisons test; Fig. 6*B*). Cumulative analysis showed that overall, VGLUT3^–/–^ mice have a higher LI than VGLUT3^+/+^ mice but that both groups show significant positive LI (Fig. 6*C*). These findings suggest that VGLUT3^–/–^ mice properly learn to extinguish their fear, with an initial higher performance than VGLUT3^+/+^ mice.

On day 15, mice were re-exposed to the original square context and their fear memory was examined (Fig. 6*D*,*E*, Recall 1). We observed a main effect of time and an interaction between time and genotype but no main effect of genotype (Table 7). *Post hoc* analysis revealed a significant difference between the freezing level of VGLUT3^–/–^ mice and VGLUT3^+/+^ mice for the first CS presentations (L2 *t*_(12)_ = 2.971, *p* = 0.03; L3 *t*_(12)_ = 3.773, *p* = 0.002 and L4 *t*_(12)_ = 2.859, *p* = 0.04; Sidak’s multiple comparisons test; Fig. 6*D*). This effect was confirmed when the first five recall sessions were analyzed separately from the last five sessions (L1–5, *t*_(12)_ = 4.076, *p* = 0.0004; L6–10, *t*_(44)_ = 1.292, *p* = 0.36; Sidak’s multiple comparisons test; Fig. 6*E*).

To establish that the freezing behavior observed during recall 1 was specific and was because of the occurrence of the light in the conditioning context, half of the animals were tested on day 18 in a completely new environment (Fig. 6*F*, Recall 2). As can be seen from Figure 6*F*, we observed no main effect of genotype or time and no interaction between time and genotype (All corresponding statistics are presented in Extended Data Figure 6-1). Freezing levels were similar (≈20–25%) for both groups, showing no evidence of generalized freezing behavior after extinction. These data suggest that after an extinction procedure, VGLUT3^–/–^ mice may have stronger original memory recall, with no generalized freezing responses to a new context.

### Working memory is intact in the absence of VGLUT3

**Figure 7.**
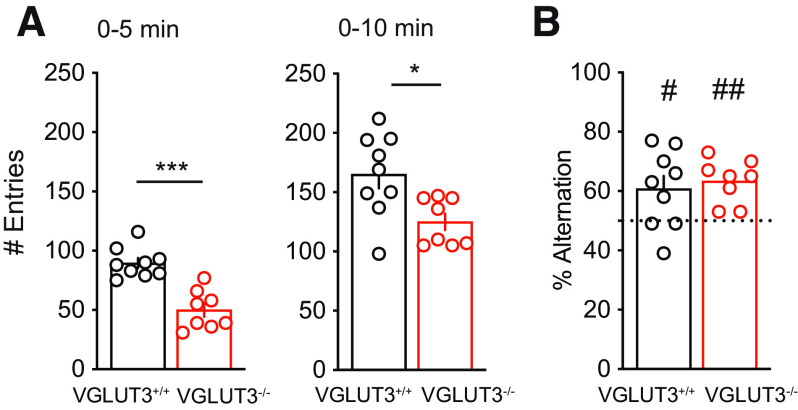
Working memory in VGLUT3^–/–^ mice. ***A***, Number of entries in the Y maze arms for the first 5 min of the test (0–5 min) or the total 10-min test (0–10 min). ***B***, Percentage of alternation. Data are mean ± SEM. Differences between genotype: *p < 0.05, ***p < 0.001. Differences compared with chance level: #*p* < 0.05, ##*p* < 0.01. All corresponding statistics are presented in Extended Data Figure 7-1.

The accelerated extinction observed in VGLUT3^–/–^ mice during the first days of extinction (Fig. 6*A*,*B*) could reflect altered working memory (WM). Hence, we compared WM of WT mice and VGLUT3^–/–^ mice using the Y maze paradigm. Mice were free to explore the Y maze for 10 min, and spontaneous alternation was quantified. In line with their anxiety phenotype, VGLUT3^–/–^ mice made significantly fewer arm entries than controls (Fig. 7*A*; All corresponding statistics are presented in Extended Data Figure 7-1). However, both groups showed similar levels of spontaneous alternation, both above chance level (Fig. 7*B*). Overall, VGLUT3^–/–^ mice show normal working memory despite a lower exploration activity.

10.1523/ENEURO.0304-22.2023.f7-1 Extended Data Figure 7-1 Statistics for the Y maze experiment. Download Figure 7-1, DOCX file.

## Discussion

The involvement of the VGLUT3 network in cognition and related psychiatric pathologies has been investigated in a few studies (Amilhon et al., 2010; Sakae et al., 2015; Balázsfi et al., 2018; Fazekas et al., 2019; Favier et al., 2020). For example, Balázsfi (2018) and Fazekas (2019) who focused on the study of learning and memory, concluded that the deficits in VGLUT3-deficient mice were very limited and mainly related to stress. Understanding how aversive memories are processed in the brain will help to decipher its dysfunction in trauma-related and stress-related disorders. In the present study we explored the establishment and maintenance of fear-related memories in mice lacking VGLUT3. Using a Pavlovian fear conditioning paradigm, we report that VGLUT3^–/–^ mice express more stable and generalized contextual memories associated with a deficit of pattern separation. Interestingly, VGLUT3^–/–^ mice have no deficit in nonaversive learning and memory, including working memory, spatial reference memory, and cue-based extinction learning. Our results partly confirm previous findings (Balázsfi et al., 2018; Fazekas et al., 2019) while deepening our understanding of the involvement of VGLUT3-dependent co-transmission in aversive memories.

Before studying aversive memories, we first assessed the consequences of the lack of VGLUT3 in learning, memory processing and cognitive flexibility in spatial and nonspatial tasks. No deficit was found in VGLUT3-deficient mice. Our results are in agreement with data obtained by Fazekas et al. (2019), who also found comparable spatial learning capacities in VGLUT3-/- mice (although they trained only male mice, in a pool that was half the size of ours), supporting the robustness of the observed phenotypes. However, our approach of systematically testing spatial memories has revealed improved long-term spatial memory performance in VGLUT3^–/–^ mice compared with control mice at 22°C in the watermaze task. Since memory performances of VGLUT3^–/–^ mice were comparable to controls when the water temperature was dropped to 19°C, we hypothesized that the improved memory performance of VGLUT3^–/–^ mice could be related to their anxiety trait (Amilhon et al., 2010) as well as to their hypothalamic-pituitary-adrenal axis dysfunctions (Balázsfi et al., 2018) in less-stressful watermaze conditions (i.e., at 22°C). This is in agreement with the literature in both humans and animals, highlighting that mild stress could have facilitating effects on memory consolidation (Sandi et al., 1997; Cahill and McGaugh, 1998; Sandi and Pinelo-Nava, 2007).

Nevertheless, depending on the behavioral paradigm used, this anxiety trait could interfere with appropriate data interpretation. In order to overcome this and accurately assess recognition memory (object and spatial) using an open-field, we had to adapt the protocol to ensure sufficient exploration of objects for recognition memory to occur. By using a fixed exploration time per session rather than a fixed session duration, we were able to circumvent the confounding effect of anxiety and ensure an unbiased assessment of recognition memory in VGLUT3^–/–^ mice. We observed no deficit of recognition or spatial memories in VGLUT3^–/–^ mice. In conclusion, using different protocols or paradigms, we confirmed that the absence of VGLUT3 does not impair spatial reference, nonspatial memory or associative-learning processes.

We next explored fear-related memories in VGLUT3^–/–^ mice using a Pavlovian fear conditioning paradigm. Because VGLUT3 is present in peripheral sensory neurons and contributes to mechanical pain detection (Seal et al., 2009), we assessed sensitivity to electric footshocks in VGLUT3^–/–^ mice and found unchanged sensitivity to electric foot shock in VGLUT3^+/+^ mice. This result confirmed previous findings by Balázsfi et al. (2018) using flinch and jump threshold as the readout. In the Pavlovian fear conditioning protocols used, the deletion of VGLUT3 led to normal fear learning but a higher and persistent contextual memory, which is consistent with observations previously published (Balázsfi et al., 2018). However, our study highlighted an absence of cue memory, which could be explained by a contextual generalization deficit. To express fear when it is relevant, present and past associations have to be compared. This is adaptive, since it allows individuals to anticipate a threat by discerning pertinent cues in the environment. Increased interference between past and new memories could promote reactivation of traumatic memories and lead to overgeneralization of fear. Considerable evidence from the literature suggests the involvement of the hippocampal CA3-dentate gyrus (DG) circuit in contextual discrimination (Cravens et al., 2006; McHugh et al., 2007; Besnard and Sahay, 2016). CA3 plays a major role in a process called pattern completion, which allows retrieval of a stored representation based on sparse cues in the environment. In contrast, the DG is also involved in pattern separation, to minimize the overlap between two similar representations. Precise memory requires remembering details with high specificity, so that memories can be discriminated from other similar memories to avoid interference. Pattern separation facilitates this discrimination by reducing the degree of similarities between overlapping experiences. The pattern separation paradigm (Sahay et al., 2011) was used to study the propensity of mice to discriminate among similar experiences (Yassa and Stark, 2011). At day 1, freezing levels were comparable between contexts A and B for both control and VGLUT3^–/–^ mice, suggesting the degree of similarity between the two contexts was high enough to evoke generalization of contextual fear in both groups. However, control mice learned to discriminate the shocked context from the safe context as early as day 7, whereas VGLUT3-deficient mice were unable to discriminate between the two contexts within the 10-d protocol used. These results highlight a significant deficit in pattern separation in VGLUT3^–/–^ mice (Fig. 4*A2*,*A3*). Considerable evidence supports a role for the hippocampus in pattern separation to constrain the overgeneralization of fear. Previous work studied the hippocampal plasticity in VGLUT3-deficient mice (Fasano et al., 2017) and found that the absence of glutamate released by VGLUT3 hippocampal interneurons led to increased GABAergic transmission, altering the oscillatory activity of synchronized networks and inducing a metaplastic shift of synaptic plasticity in the ventral hippocampus. As hippocampal long-term plasticity is currently thought to underlie the cellular basis of such learning and memory processes, we cannot exclude that they might cause the observed contextual overgeneralization in VGLUT3^–/–^ mice.

To better understand this discrimination deficit, we performed an immediate shock (IS) test. According to Fanselow (2000), in the IS test, animals do not have enough time to form an integrated memory representation of context features to associate it to the electric shock. In line with this hypothesis, wild-type mice do not form a contextual fear memory and show no freezing behavior during retrieval tests. In contrast, when they were immediately shocked, VGLUT3^–/–^ mice increased their level of freezing whatever the context used in the retrieval test (Fig. 5). This observation suggests that in VGLUT3^–/–^ mice, the mere occurrence of the traumatic event (i.e., the foot shock) elicited impaired fear expression. In our view, this increased fear expression reflects more than innate fear impairment in VGLUT3^–/–^ mice, since innate fear experiences to natural threats need to be harmless (Silva et al., 2016). When the animal experiences pain such as a foot shock as in our experiment, it is a conditioned response and a learned experience.

One major treatment of fear-related disorders, called exposure therapy in clinics or extinction fear learning in laboratory, involves repeatedly re-exposure of animals to the CS (the flashing light) previously associated with the aversive US (the foot shock) in a different context. With time, the animals learn that the CS is no longer associated with the US in this new context and thus the mice form a new “safer” memory (Myers and Davis, 2007; Perusini and Fanselow, 2015). Surprisingly, during the initial steps of this extinction learning, VGLUT3^–/–^ mice show improved performance (Fig. 6*A–C*). This is particularly surprising since the processes governing extinction and generalization are thought to be similar (see Lopresto et al., 2016). The brain structures mainly involved are the prefrontal cortex [especially its infra limbic (IL-PFC) part] and the hippocampus as previously discussed regarding pattern separation. However, extinction mostly relies on the interaction of the IL-PFC with the basal-lateral amygdala. Those projections do not express VGLUT3 and might effectively control the amygdala activity as observed. One hypothesis is that context generalization (or lack of pattern separation) could be because of the dysfunction of the hippocampal network because of the absence of VGLUT3, whereas the cue-based extinction may depend on the IL-PFC projections to the amygdala.

Original memory was assessed at day 15 in the conditioning context (Fig. 6*D*,*E*). Results confirm that the extinction procedure did not alter the original memory since both groups still displayed a high level of freezing (significantly higher in VGLUT3^–/–^ than in control mice) to the context where they were originally shocked. Surprisingly, when tested in a third context on D18 VGLUT3^–/–^ mice did not show fear generalization, indicating that the animals might have associated the aversive value of the CS only to the original context. This observation suggests that VGLUT3^–/–^ mice could show an associative cue learning that can properly be recalled and specific to a context.

In regards to the initial facilitation of the extinction, we cannot exclude that this could be because of increased attention related to the anxiety trait in VGLUT3^–/–^ mice, or in their working memory. Attentional processes are difficult to test in VGLUT3^–/–^ mice, since those experiments classically require the use of sound (e.g., prepulse inhibition, fear startle tests…) and these mutants are deaf (Ruel et al., 2008). To rule out any working memory modification that could explain this initial extinction improvement, we subjected our mice to a Y maze alternation protocol. Unlike Fazekas et al. (2019), we observed no alteration of working memory in VGLUT3^–/–^ mice. Since mice lacking VGLUT3 tend to explore less because of their anxious phenotype, we increased the test duration from 5 to 10 min to have substantial exploration levels in VGLUT3^–/–^ mice and WT mice (>100 entries). This might explain the different findings, since poor exploration can directly affect behavioral performances. Therefore, in our hands, VGLUT3^–/–^ mice show no deficit or facilitation of their working memory that could explain their better initial performance in fear extinction.

Some studies found VGLUT3-amacrine cells in mouse retina (Kim et al., 2015; Lee et al., 2016, 2021) co-releasing glutamate and glycine at glycinergic synapses. How the absence of VGLUT3 could impact the function of these synapses in these animals, and therefore, their ability to see properly has yet to be determined. What seems to be accepted is the lack of VGLUT3 impacting the vision of movement (Kim et al., 2015; Lee et al., 2016). However, based on our results, it is unlikely that the observed initial lack of cue conditioning can be because of visual impairment. First, we use a flashing light as a cue, that is a major visual information. Then, VGLUT3-deficient mice have intact performances in the spatial reference memory task in the watermaze, and in the object recognition tasks, both of which mainly rely on visual cues. Overall, we cannot rule out differences in visual detection between controls and VGLUT3-deficient mice, but this alone cannot explain the initial lack of cue conditioning observed.

Our findings on impaired fear-related memories in mice lacking VGLUT3 are in good agreement with the electrophysiological reports (Fasano et al., 2017). However, this interpretation should be taken with care, since a constitutive VGLUT3 deletion was used in the present study. Cholinergic fibers from the basal forebrain projecting to the basolateral amygdala are crucial in reinforcing learning and consolidating aversive memories (Jiang et al., 2016; Aitta-Aho et al., 2018; Crouse et al., 2020). Interestingly, a subset of those fibers does express VGLUT3 (Nickerson Poulin et al., 2006). It is possible that this cholinergic pathway could also be involved in fear-related disorders. A thorough description of the involvement of these different pathways would require the deletion of VGLUT3 in specific subpopulation of neurons.

In conclusion, the present study suggests an important role of VGLUT3 in aversive memory processing such as contextual generalization of fear memory which could be crucial in trauma-related and stress-related disorders.

## Synthesis

Reviewing Editor: Erica Glasper, The Ohio State University Decisions are customarily a result of the Reviewing Editor and the peer reviewers coming together and discussing their recommendations until a consensus is reached. When revisions are invited, a fact-based synthesis statement explaining their decision and outlining what is needed to prepare a revision will be listed below. The following reviewer(s) agreed to reveal their identity: Dora Zelena, Patrick Piantadosi.

**Manuscript Synthesis**: In this manuscript, the authors aimed to determine the consequences of vesicular glutamate transporter type 3 (Vglut3) deletion on affective and cognitive behaviors in mice. It has previously been demonstrated that Vglut3 deletion results in stress hyperreactivity, HPA-axis dysfunction, and alterations in conditioned fear response, shock sensitivity, and behavior within the Morris water maze and y-maze tasks. Despite this knowledge, far less is known about the contribution of Vglut3 to stress-related behaviors, including multiple facets of fear learning (e.g., acquisition, generalization, and extinction). Using Vglut3 KO mice, the authors demonstrate that Vglut3 depletion leads to elevated contextual fear and a loss of pattern separation that results in elevated fear-like behavior in contexts not associated with shock. Additionally, the authors demonstrate that one-trial fear learning is present in Vglut3 KO mice - an effect not observed in WT mice. This further suggests that context learning is enhanced in Vglut3 KO mice; cued fear conditioning also extinguishes more rapidly in Vglut3 KO mice compared to WT mice. In addition to these behavioral observations, the authors utilize necessary controls to show that deletion of Vglut3 does not alter other physiological and psychological processes. The reviewers found this study of high importance, and will be of great interest to the field, there are design, interpretation, and conceptual concerns that should be addressed (itemized below). Overall, the manuscript would benefit from a significant grammatical revision. Additionally, as you can see, there are numerous minor comments that need to be addressed to provide greater clarity within the methods and results section. Where appropriate, individual reviewer comments have been included and *italicized*.

**Major Comments**

1. The experiments within this manuscript do not support the mechanism mentioned in the manuscript title. This is restated many times within the text and should be supported by citations or data generated by the authors.

2. Since prior studies have utilized this strain of Vglut3 KO mice, this manuscript may be of greater value if the authors could focus their attention on specific brain areas in Cre mice strains that were mentioned in the Introduction.

3. In this manuscript, the authors aimed to connect fear and post-traumatic stress disorder (PTSD), however, mice were behaviorally assessed 24h following shock (or at least started to study them). This is more aligned with an acute stress response rather than a long-term effect, which is more comparable to clinical indices of PTSD. The authors should see the DSM-V criteria of PTSD and address this apparent discrepancy.

4. The authors should elucidate the conceptual differences between pattern separation, immediate shock, and fear extinction. For example, *“immediate shock examines generalization of the freezing (in novel box), but Context B in pattern separation paradigm does almost the same (or more visual perception???). Moreover, during repeated testing in pattern separation the extinction was also studied. Furthermore, during fear extinction “in the modified context” (whatever does it mean) was used, which is questionable as “normally” Context A is used for extinction training.”*

5. Please provide more detail regarding the pattern separation experiment. For example, why did the control mice diverge so dramatically on D7. *Do the authors have any thoughts about why pattern separation emerges spontaneously in such a way?*

6. Please explain why female mice were used for the MWM experiments, but not for others.

7. Given that the authors have presented statistics in the Tables, it is not necessary to include this information in the Results section as well. Some of the information in the current Results section include sentences that would be better suited in the Introduction, Methods, or even Discussion sections.

8. In many instances, p-values are reported but it is not clear what groups are being compared. If Sidak’s multiple comparisons tests were performed for post-hoc analysis, then this p-value should be reported, and groups being compared should be explicitly stated. Furthermore, if an interaction or main effect is observed, the authors must provide greater detail regarding what factors are being discussed. Here is an example: we observed an interaction between X and Y, but no main effect of X and no main effect of Y.

9. The Discussion sections requires significant editing. The first paragraph should be a summary of the findings. In some instances, the paragraphs include what appears to be a loose collection of sentences, rather than an analysis of the literature as it relates to the current findings. For example, Balázsfi et al 2018 and Fazekas et al 2019 used all the behavioral tests presented here in VGluT3 KO mice. Also, incomplete paragraphs, thoughts, and sentences are found throughout. The authors are encouraged to move relevant discussions of the data, currently found in the Result section, to the Discussion section.

10. It is unclear why the paragraph starting with “The basolateral amygdala (BLA) and the hippocampus” was included in the Discussion. This brain region was not examined in the present study. Greater emphasis should be given to the specific tests, and their different aspects (generalization, discrimination, cognitive flexibility, extinction etc.) and how they relate to existing findings in the literature as they relate to Vglut3.

11. The main conclusion of the paper is not supported by the data. The authors are encouraged to temper their interpretation of their data.

**Minor Comments**

1. The authors are encouraged to emphasize the fact that this study was designed to confirm prior results, while also explaining that similar results are expected despite differences in labs or animal facilities. Doing so will demonstrate that the observed outcomes are a result of the deletion of Vglut3 as opposed to other experimental differences.

2. Balázsfi et al 2018 assessed fear using a contextual fear conditioning paradigm. Please revise the discussion of this paper.

3. In what country can the “Ministère de l’Agriculture et de la Forêt, Service Vétérinaire de la Santé et de la Protection Animale” be found?

4. Please provide greater detail regarding the Vglut3 KO mice.

a. *When were the mice genotyped?*

b. *Were their digits cut to mark them?*

5. Please clarify what “CS went off” means. Does this mean that *a flashing light was switched on, which was switched off as CS?*

6. Please report whether the different water temperatures directly affected learning.

7. Please report the intertrial interval for the following phrase: “4 CS presentations with an interval of 30 sec”.

8. Please provide greater detail for this phrase: “during the first 180 sec for each context.” Specifically, *how much time did the mice spent in the context?*

9. Y-maze should be included in the description of spatial and non-spatial memory tests.

10. Page 8, first results paragraph: The first sentence should be revised, as it includes mention of novel object recognition task. These data were not reported in this paragraph. Also, in this same paragraph, to compare WT littermates and Vglut3 KO mice probe trial time in the target quadrant, please perform a 2-tailed t-test.

11. Please define what “NPR” refers to.

12. Please add a schematic to Figure 1.

13. Figure 2 (and others): What do ** and *** refer to statistically? Do they reflect significant differences from 50% measured by 2-tailed sample t-tests?

14. Please revise the x-axis in Figure 3, so that is it *harmonized to be in minutes throughout (the bar graph reports time bins of 0-3 min and 3-6 min, whereas the line graphs are needlessly in seconds).*

15. The authors should include exploration time during the 10-min NOR and NPR tests. It is important to report whether Vglut3 KO mice explored the objects to the same extent as the WT mice.

16. The following sentence is unnecessary, as it was previously clarified: “Since VGLUT3-/- mice are deaf (Ruel et al., 2008; Seal et al., 2008), it was not possible to use the classical light-electric shock association to induce fear conditioning. Therefore, sound was replaced by a flashing light (see methods) coupled to an electric footshock (US).”

17. The interpretation of the following statement should be reconsidered: “These results suggest that VGLUT3-/- mice might have a more persistent contextual memory than WT mice, or a deficit in extinction processes.” Could this simply be an anxiogenic response?

a. While the interaction factors aren’t explicitly stated, the presumptive genotype X time interaction suggests that the genotypes have different time curves.

b. Please consider, as displayed on the figures, the use of 1-minute bins. 3-minute bins do not provide useful information.

18. Please consider that the lack of a cue effect might be the result of a visual deficit, as some Vglut3 KO mice have retinal issues (e.g., DOI:10.1113/JP281717).

19. Please move the following sentence to the methods section to increase clarity: “However, context A is always associated with an electric shock (ES), whereas context B is safe and free of ES”.

20. The following requires more information: “they significantly freeze less from Day 7 to Day 10”. The addition of “*in Context B than in shock-associated Context A*.” may improve clarity.

21. The following may be related to visual impairments and should be considered: “VGLUT3-/- mice present a deficit in pattern separation” and “These findings suggest that VGLUT3-/- mice properly learn a discrete CS-US association as well as its extinction, with an initial higher performance than VGLUT3+/+ mice. Therefore, the constitutive invalidation of VGLUT3 seems to facilitate extinction of fear memory, and do not prevent discrete CS-US association.”

22. This statement is in contrast of a previous statement suggesting Vglut3 promotes generalization of fear: “These data suggest that VGLUT3-/- mice may have a stronger original memory, and that the extinction procedure is selective of the context in which the extinction occurred and does not lead to generalized freezing responses to new context.”
