# Supplementary material for: Absence of VGLUT3 Expression Leads to Impaired Fear Memory in Mice
Source: eNeuro. 2023 Feb 22;10(2):ENEURO.0304-22.2023. doi: 10.1523/ENEURO.0304-22.2023 (PMC9953049; doi:10.1523/ENEURO.0304-22.2023)
Supplement: Extended Data Figure 1-1 — Statistics for watermaze experiments. 1: SRM 10 min; 2: SRM 72 h; 3: SRM-R 10 min; 4: SRM-R 48 h; 5: PTs average. Download Figure 1-1, DOCX file. [file enu-eN-NWR-0304-22-s02.docx]

| Figure 1 | N (mice) | Statistical analysis | | value | p-value |
| --- | --- | --- | --- | --- | --- |
| Fig. 1D | WT (n=15), KO (n=11) | Two-way RM ANOVA | Genotype | F_1,24_=0.5923 | 0.4490 |
|  |  |  | Days | F_3,72_=79.33 | **<0.0001** |
|  |  |  | Genotype x Days | F_3,72_=0.9438 | 0.4241 |
| Fig. 1E |  | Two-way RM ANOVA | Genotype | F_1,24_=0.00026 | 0.9871 |
|  |  |  | Days | F_7,168_=50.24 | **<0.0001** |
|  |  |  | Genotype x Days | F_7,168_=0.9339 | 0.4819 |
| Fig. 1F^1^ |  | Unpaired t test (two-tailed) | | t=1.44, df=24 | 0.1607 |
|  |  | WT to 25% chance level | | t=6.853, df=14 | **<0.0001** |
|  |  | KO to 25% chance level | | t=7.007, df=10 | **<0.0001** |
| Fig. 1F^2^ |  | Unpaired t test (two-tailed) | | t=2.60, df=24 | **0.0156** |
|  |  | WT to 25% chance level | | t=5.913, df=14 | **<0.0001** |
|  |  | KO to 25% chance level | | t=11.48, df=10 | **<0.0001** |
| Fig. 1F^3^ |  | Unpaired t test (two-tailed) | | t=1.16, df=24 | 0.2543 |
|  |  | WT to 25% chance level | | t=4.542, df=14 | **0.0005** |
|  |  | KO to 25% chance level | | t=6.451, df=10 | **<0.0001** |
| Fig. 1F^4^ |  | Unpaired t test (two-tailed) | | t=1.45, df=24 | 0.1601 |
|  |  | WT to 25% chance level | | t=4.069, df=14 | **0.0011** |
|  |  | KO to 25% chance level | | t=7.218, df=10 | **<0.0001** |
| Fig. 1F^5^ |  | Unpaired t test (two-tailed) | | t=2.38, df=24 | **0.0254** |
|  |  | WT to 25% chance level | | t=7.789, df=14 | **<0.0001** |
|  |  | KO to 25% chance level | | t=11.35, df=10 | **<0.0001** |
| Fig. 1G | WT (n=13), KO (n=11) | Two-way RM ANOVA | Genotype | F_1,22_=0.0666 | 0.7987 |
|  |  |  | Days | F_3,66_=118.3 | **<0.0001** |
|  |  |  | Genotype x Days | F_3,66_=0.4520 | 0.7167 |
| Fig. 1H |  | Two-way RM ANOVA | Genotype | F_1,22_=0.0026 | 0.9592 |
|  |  |  | Days | F_7,154_=17.94 | **<0.0001** |
|  |  |  | Genotype x Days | F_7,154_=0.6502 | 0.7138 |
| Fig. 1I^1^ |  | Unpaired t test (two-tailed) | | t=0.96, df=22 | 0.3462 |
|  |  | WT to 25% chance level | | t=8.747, df=12 | **<0.0001** |
|  |  | KO to 25% chance level | | t=5.790, df=10 | **0.0002** |
| Fig. 1I^2^ |  | Unpaired t test (two-tailed) | | t=0.50, df=22 | 0.6193 |
|  |  | WT to 25% chance level | | t=4.101, df=12 | **0.0015** |
|  |  | KO to 25% chance level | | t=3.807, df=10 | **0.0034** |
| Fig. 1I^3^ |  | Unpaired t test (two-tailed) | | t=0.01, df=22 | 0.9872 |
|  |  | WT to 25% chance level | | t=4.277, df=12 | **0.0011** |
|  |  | KO to 25% chance level | | t=4.405, df=10 | **0.0013** |
| Fig. 1I^4^ |  | Unpaired t test (two-tailed) | | t=0.12, df=22 | 0.9016 |
|  |  | WT to 25% chance level | | t=3.728, df=12 | **0.0029** |
|  |  | KO to 25% chance level | | t=6.588, df=10 | **<0.0001** |
| Fig. 1I^5^ |  | Unpaired t test (two-tailed) | | t=0.51, df=22 | 0.6126 |
|  |  | WT to 25% chance level | | t=9.224, df=12 | **<0.0001** |
|  |  | KO to 25% chance level | | t=7.347, df=10 | **<0.0001** |
| Fig. 1F/I | WT-22°C (n=15) KO-22°C (n=11) WT-19°C (n=13) KO-19°C (n=11) | 3 way ANOVA | Genotype | F_1,46_=1.641 | 0.2066 |
|  |  |  | Temperature (T) | F_1,46_=0.51 | 0.4786 |
|  |  |  | Probe Test (PT) | F_3,138_=1.12 | 0.3433 |
|  |  |  | Genotype x PT | F_3,138_=0.1064 | 0.9562 |
|  |  |  | Genotype x T | F_1,46_=4.09 | **0.04** |
|  |  |  | T x PT | F_3,138_=0.5871 | 0.6244 |
|  |  |  | Genotype  x T x PT | F_3,138_=0.2556 | 0.8573 |

^1^: SRM 10min ; ^2^: SRM 72h ; ^3^: SRM-R 10min ; ^4^: SRM-R 48h ; ^5^: PTs average
