# Supplementary material for: Absence of VGLUT3 Expression Leads to Impaired Fear Memory in Mice
Source: eNeuro. 2023 Feb 22;10(2):ENEURO.0304-22.2023. doi: 10.1523/ENEURO.0304-22.2023 (PMC9953049; doi:10.1523/ENEURO.0304-22.2023)
Supplement: Extended Data Figure 2-1 — Statistics for object recognition experiments. Download Figure 2-1, DOCX file. [file enu-eN-NWR-0304-22-s03.docx]

| **Figure 2** | **N (mice)** | **Statistical analysis** | **value** | **p-value** |
| --- | --- | --- | --- | --- |
| Fig. 2A | WT (n=13) | One sample t test (to 0.5) | t=5.64, df=12 | **0.0001** |
|  | KO (n=12) | One sample t test (to 0.5) | t=6.84, df=11 | **<0.0001** |
|  | WT (n=13), KO (n=12) | Mann-Whitney test | U=35 | **0.0188** |
| Fig. 2B | WT (n=13) | One sample t test (to 0.5) | t=15.39, df=12 | **<0.001** |
|  | KO (n=12) | One sample t test (to 0.5) | t=4.33, df=11 | **0.0012** |
|  | WT (n=12), KO (n=13) | Mann-Whitney test | U=76 | 0.936 |
| Fig. 2C | WT (n=13) | Simple Linear Regression | R2=0.44  F(1,10)=8.66 | **0.0134** |
|  | KO (n=12) | Simple Linear Regression | R2=0.0299  F(1,11)=0.3084 | 0.59 |
