# Supplementary material for: Absence of VGLUT3 Expression Leads to Impaired Fear Memory in Mice
Source: eNeuro. 2023 Feb 22;10(2):ENEURO.0304-22.2023. doi: 10.1523/ENEURO.0304-22.2023 (PMC9953049; doi:10.1523/ENEURO.0304-22.2023)
Supplement: Extended Data Figure 3-1 — Statistics for fear conditioning experiments. Download Figure 3-1, DOCX file. [file enu-eN-NWR-0304-22-s04.docx]

| **Figure 3** | **N (mice)** | **Statistical analysis** | | **value** | **p-value** |
| --- | --- | --- | --- | --- | --- |
| Fig. 3A | WT (n=8), KO (n=6) | Two-way RM ANOVA | Genotype | F_1,12_=0.6328 | 0.4418 |
|  |  |  | Behaviors | F_3,36_=36.57 | **<0.0001** |
|  |  |  | Genotype x Behaviors | F_3,36_=3.874 | **0.0169** |
| Fig. 3B | WT (n=12), KO (n=12) | Two-way RM ANOVA | Genotype | F_1,22_=1.540 | 0.2277 |
|  |  |  | Time | F_7,154_=14.88 | **<0.0001** |
|  |  |  | Genotype x Time | F_7,154_=0.8944 | 0.5125 |
| Fig. 3C |  | Two-way RM ANOVA | Genotype | F_1,22_=2.602 | 0.1210 |
|  |  |  | Time | F_11,242_=4.012 | **<0.0001** |
|  |  |  | Genotype x Time | F_11,242_=4.161 | **<0.0001** |
| Fig. 3D |  | Two-way RM ANOVA | Genotype | F_1,22_=2.602 | 0.1210 |
|  |  |  | Time | F_1,22_=5.271 | **0.0316** |
|  |  |  | Genotype x Time | F_1,22_=5.775 | **0.0251** |
| Fig. 3E |  | Two-way RM ANOVA | Genotype | F_1,22_=1.496 | 0.2343 |
|  |  |  | Time | F_11,242_=3.554 | **0.0001** |
|  |  |  | Genotype x Time | F_11,242_=0.8698 | 0.5707 |
| Fig. 3F |  | Two-way RM ANOVA | Genotype | F_1,22_=1.32 | 0.2629 |
|  |  |  | Time | F_1,22_=7.743 | **0.0109** |
|  |  |  | Genotype x Time | F_1,22_=0.6567 | 0.4264 |
