# Supplementary material for: Absence of VGLUT3 Expression Leads to Impaired Fear Memory in Mice
Source: eNeuro. 2023 Feb 22;10(2):ENEURO.0304-22.2023. doi: 10.1523/ENEURO.0304-22.2023 (PMC9953049; doi:10.1523/ENEURO.0304-22.2023)
Supplement: Extended Data Figure 4-1 — Statistics for pattern separation experiment. Download Figure 4-1, DOCX file. [file enu-eN-NWR-0304-22-s05.docx]

| **Figure 4** | **N (mice)** | **Statistical analysis** | | **value** | **p-value** |
| --- | --- | --- | --- | --- | --- |
| Fig. 4B | WT (n=11) | Two-way RM ANOVA | Context | F_1,20_=2.800 | 0.1098 |
|  |  |  | Time | F_9,180_=22.68 | **<0.0001** |
|  |  |  | Context x Time | F_9,180_=9.302 | **<0.0001** |
| Fig. 4C | KO (n=10) | Two-way RM ANOVA | Context | F_1,18_=0.8957 | 0.3565 |
|  |  |  | Time | F_9,162_=3.823 | **0.0002** |
|  |  |  | Context x Time | F_9,162_=0.8542 | 0.5675 |
| Fig. 4D | WT (n=11), KO (n=10) | Mann-Whitney test | WT vs. KO | U=44 | 0.2143 |
| Fig. 4E |  | Two-way RM ANOVA | Genotype | F_1,19_=18.40 | **0.0004** |
|  |  |  | Context | F_1,19_=2.38 | 0.1394 |
|  |  |  | Genotype x Context | F_1,19_=0.819 | 0.3768 |
| Fig. 4F |  | Two-way RM ANOVA | Genotype | F_1,19_=0.9829 | 0.3339 |
|  |  |  | Context | F_1,19_=5.490 | **0.0302** |
|  |  |  | Genotype x Context | F_1,19_=10.45 | **0.0044** |
| Fig. 4G |  | Two-way RM ANOVA | Genotype | F_1,19_=0.9582 | 0.3399 |
|  |  |  | Context | F_1,19_=27.96 | **<0.0001** |
|  |  |  | Genotype x Context | F_1,19_=32.06 | **<0.0001** |
