# Supplementary material for: Absence of VGLUT3 Expression Leads to Impaired Fear Memory in Mice
Source: eNeuro. 2023 Feb 22;10(2):ENEURO.0304-22.2023. doi: 10.1523/ENEURO.0304-22.2023 (PMC9953049; doi:10.1523/ENEURO.0304-22.2023)
Supplement: Extended Data Figure 5-1 — Statistics for immediate shock experiments. NS: no shock; IS: immediate shock; SC: same context; NC: new context Download Figure 5-1, DOCX file. [file enu-eN-NWR-0304-22-s06.docx]

| **Figure 5** | **N (mice)** | **Statistical analysis** | | **value** | **p-value** |
| --- | --- | --- | --- | --- | --- |
| Fig. 5 | WT  (NS, n=7),  (IS-SC, n=7),  (IS-NC, n=8).    KO  (NS, n=10),  (IS-SC, n=10),  (IS-NC, n=8). | Two-way ANOVA | Genotype | F_1,43_=24.34 | **<0.0001** |
|  |  |  | Group | F2_,43_=5.198 | **0.0095** |
|  |  |  | Genotype x Group | F_2,43_=3.592 | **0.0361** |
|  |  | Sidack’s MC | NS:WT vs. NS:KO | t=0.76, df=43 | 0.9999 |
|  |  | Sidack’s MC | NS:WT vs. IS-SC:WT | t=0.36, df=43 | >0.9999 |
|  |  | Sidack’s MC | NS:WT vs. IS-SC:KO | t=4.79, df=43 | **0.0003** |
|  |  | Sidack’s MC | NS:WT vs. IS-NC:WT | t=0.19, df=43 | >0.9999 |
|  |  | Sidack’s MC | NS:WT vs. IS-NC:KO | t=3.57, df=43 | **0.0135** |
|  |  | Sidack’s MC | NS:KO vs. IS-SC:WT | t=0.37, df=43 | >0.9999 |
|  |  | Sidack’s MC | NS:KO vs. IS-SC:KO | t=4.44, df=43 | **0.0009** |
|  |  | Sidack’s MC | NS:KO vs. IS-NC:WT | t=0.55, df=43 | >0.9999 |
|  |  | Sidack’s MC | NS:KO vs. IS-NC:KO | t=3.10, df=43 | **0.0499** |
|  |  | Sidack’s MC | IS-SC:WT vs. IS-SC:KO | t=4.40, df=43 | **0.0011** |
|  |  | Sidack’s MC | IS-SC:WT vs. IS-NC:WT | t=0.17, df=43 | >0.9999 |
|  |  | Sidack’s MC | IS-SC:WT vs. IS-NC:KO | t=3.20, df=43 | **0.0390** |
|  |  | Sidack’s MC | IS-SC:KO vs. IS-NC:WT | t=4.58, df=43 | **0.0006** |
|  |  | Sidack’s MC | IS-SC:KO vs. IS-NC:KO | t=1.09, df=43 | 0.9931 |
|  |  | Sidack’s MC | IS-NC:WT vs. IS-NC:KO | t=3.36, df=43 | **0.0242** |
