# Supplementary material for: Absence of VGLUT3 Expression Leads to Impaired Fear Memory in Mice
Source: eNeuro. 2023 Feb 22;10(2):ENEURO.0304-22.2023. doi: 10.1523/ENEURO.0304-22.2023 (PMC9953049; doi:10.1523/ENEURO.0304-22.2023)
Supplement: Extended Data Figure 6-1 — Statistics for fear extinction experiments. Download Figure 6-1, DOCX file. [file enu-eN-NWR-0304-22-s07.docx]

| **Figure 6** | **N (mice)** | **Statistical analysis** | | **value** | **p-value** |
| --- | --- | --- | --- | --- | --- |
| Fig. 6A | WT (n=12), KO (n=12) | Two-way RM ANOVA | Genotype | F_1,22_=3.520 | 0.0740 |
|  |  |  | Time | F_69,1518_=12.25 | **<0.0001** |
|  |  |  | Genotype x Time | F_69,1518_=1.813 | **<0.0001** |
| Fig. 6B |  | Two-way RM ANOVA | Genotype | F_1,22_=6.395 | **0.0191** |
|  |  |  | Time | F_6,132_=1.061 | 0.3895 |
|  |  |  | Genotype x Time | F_6,132_=1.543 | 0.1690 |
| Fig. 6C |  | Unpaired t-test | WT vs. KO | t=2.529 df=22 | **0.0191** |
|  |  | Wilocoxon | WT vs. LI=0 | W=66 | **0.006** |
|  |  |  | KO vs. LI=0 | W=78 | **0.0005** |
| Fig. 6D |  | Two-way RM ANOVA | Genotype | F_1,22_=2.899 | 0.1027 |
|  |  |  | Time | F_9,198_=21.30 | **<0.0001** |
|  |  |  | Genotype x Time | F_9,198_=4.828 | **<0.0001** |
| Fig. 6E |  | Two-way RM ANOVA | Genotype | F_1,22_=2.899 | 0.1027 |
|  |  |  | Time | F_1,22_=103.4 | **<0.0001** |
|  |  |  | Genotype x Time | F_1,22_=21.71 | **0.0001** |
