# Supplementary material for: Absence of VGLUT3 Expression Leads to Impaired Fear Memory in Mice
Source: eNeuro. 2023 Feb 22;10(2):ENEURO.0304-22.2023. doi: 10.1523/ENEURO.0304-22.2023 (PMC9953049; doi:10.1523/ENEURO.0304-22.2023)
Supplement: Extended Data Figure 7-1 — Statistics for the Y maze experiment. Download Figure 7-1, DOCX file. [file enu-eN-NWR-0304-22-s08.docx]

| **Figure 7** | **N (mice)** | **Statistical analysis** | **value** | **p-value** |
| --- | --- | --- | --- | --- |
| Fig. 7A  0-5min | WT (n=9), KO (n=8) | Mann-Whitney test | U=1 | **0.0002** |
| Fig. 7A  0-10min |  | Mann-Whitney test | U=11 | **0.0139** |
| Fig. 7B |  | Unpaired t test | t=0.4952, df=15 | 0.6276 |
| Fig. 7B | WT (n=9) | One sample t test (to 50%) | t=2.469, df=8 | **0.0387** |
| Fig. 7B | KO (n=8) | One sample t test (to 50%) | t=5.163, df=7 | **0.0013** |
